# Diverse Modes of Axon Elaboration in the Developing Neocortex

**DOI:** 10.1371/journal.pbio.0030272

**Published:** 2005-07-26

**Authors:** Carlos Portera-Cailliau, Robby M Weimer, Vincenzo De Paola, Pico Caroni, Karel Svoboda

**Affiliations:** **1**Howard Hughes Medical Institute, Cold Spring Harbor Laboratory, Cold Spring Harbor, New York, United States of America,; **2**Department of Biological Sciences, Columbia University, New York, New York, United States of America,; **3**Friedrich Miescher Institute, Basel, Switzerland; Harvard UniversityUnited States of America

## Abstract

The development of axonal arbors is a critical step in the establishment of precise neural circuits, but relatively little is known about the mechanisms of axonal elaboration in the neocortex. We used in vivo two-photon time-lapse microscopy to image axons in the neocortex of green fluorescent protein-transgenic mice over the first 3 wk of postnatal development. This period spans the elaboration of thalamocortical (TC) and Cajal-Retzius (CR) axons and cortical synaptogenesis. Layer 1 collaterals of TC and CR axons were imaged repeatedly over time scales ranging from minutes up to days, and their growth and pruning were analyzed. The structure and dynamics of TC and CR axons differed profoundly. Branches of TC axons terminated in small, bulbous growth cones, while CR axon branch tips had large growth cones with numerous long filopodia. TC axons grew rapidly in straight paths, with frequent interstitial branch additions, while CR axons grew more slowly along tortuous paths. For both types of axon, new branches appeared at interstitial sites along the axon shaft and did not involve growth cone splitting. Pruning occurred via retraction of small axon branches (tens of microns, at both CR and TC axons) or degeneration of large portions of the arbor (hundreds of microns, for TC axons only). The balance between growth and retraction favored overall growth, but only by a slight margin. Given the identical layer 1 territory upon which CR and TC axons grow, the differences in their structure and dynamics likely reflect distinct intrinsic growth programs for axons of long projection neurons versus local interneurons.

## Abstract 2

Long-term in vivo two-photon imaging of developing thalamocortical and cajal-retzius axons reveals distinct modes of axon elaboration in neocortex.

## Introduction

In the developing mammalian brain, axons elaborate to form complex arbors that innervate specific target cells. In the peripheral and central nervous systems, axons have an early phase of rapid growth, followed by a period of activity-dependent pruning [1–8]. But the notion that axons develop through sequential “growth then pruning” remains controversial [9], as axons may undergo simultaneous growth and pruning [10–12] or monotonic growth [13]. Time-lapse in vivo imaging has been critical for understanding axonal growth and refinement in lower vertebrates [14,15]. In contrast, prior studies of the developing mammalian brain have relied on measurements in fixed brain preparations. Therefore, relatively little is known about how axonal projections are refined within their target regions, at the level of a single cortical layer or column, or about the exact time course of growth and pruning. Even less is known about the elaboration of interneuron axonal arbors.

Axonal growth, guidance, and branching are key elements of axonal development. These developmental processes have been studied most thoroughly in cultured neurons. Axon elaboration involves the extension of distal tips and the formation of new processes by branching. The tips of growing axons classically exhibit elaborate growth cones with multiple filopodia [16,17]. Growth cones are thought to be critical for guidance by contact-mediated or chemical cues [18]. For example, an individual axon's growth cone morphology can vary according to whether the axon is growing rapidly along a fiber tract or pausing at a decision-making point [19–23]. It is also possible that different growth programs for different axon types are associated with distinct growth cone geometries. The exact role of the terminal growth cone in branching is also a point of contention [22,24]. Although direct (growth cone splitting [25]) and indirect (delayed branching [26]) roles of growth cones have been proposed for branching of some axons, interstitial branching independent of growth cones also plays a prominent role in axon elaboration in some cases [27]. The relative importance of these processes in the intact cortex is unknown.

Branch elimination is another prominent feature of axon development. Two general processes have been described in developing neurons in various species. In *Drosophila,* axons of mushroom body neurons are eliminated during metamorphosis by axonal degeneration [28], a process reminiscent of Wallerian degeneration [29,30]. In the mouse hippocampus, pruning is presumably manifested as axon tip retraction [31] (but see [32]). It is conceivable that different types of axons prune branches in different ways, or that these different modes of pruning may operate over different length scales [33].

To study axonal growth and pruning in the intact brain we used two-photon microscopy in transgenic mice expressing membrane-bound green fluorescent protein (GFP) under the *thy-1* promoter [34]. We imaged the elaboration of cortical axons within layer 1 during early postnatal development. We compared long-range projection axons of thalamocortical (TC) neurons with local axons of Cajal-Retzius (CR) interneurons. TC axons begin to invade the cortex around the time of birth and elaborate their arbors over the following 2–3 wk [13,35–39]. We imaged axons originating from thalamic neurons that project to barrel cortex. We focused on secondary (diffuse) thalamic projections [40–43] originating from the Pom (the medial part of the posterior nucleus of the thalamus), which target layer 5A and supragranular layers, including layer 1 [44,45]. In contrast, primary thalamic projections from the ventral posteromedial (VPm) subdivision of the ventrobasal nucleus largely target layers 4 and 5B of sensory cortex, and do not project to layer 1 [13]. Individual Pom axons project more widely than VPm axons and target complementary territories. Pom axons span multiple barrel columns in primary somatosensory cortex and often send branches to higher-order sensory areas and motor areas [44,45]. It has been suggested that diffuse thalamic projections mediate sensory-motor integration and thalamocortical synchrony [43].

CR neurons are born relatively early during embryonic life (embryonic day 10–14 [E10–E14]) [46], but their axons are still growing during the first 2 wk of postnatal life, as suggested by the presence of growth cones at their tips [47,48]. The cell bodies, dendrites and axons of CR neurons are entirely contained within layer 1. Thus, CR cells are the earliest cortical interneurons [49]. Because of the strategic location in layer 1, it has been suggested that CR neurons are important for neuronal migration and the generation of the inside-out cortical layering [46]. Indeed, CR neurons secrete the protein Reelin, which is critical for the development of cortical lamination [50,51]. CR axons synapse principally on the growing apical dendritic arbors of pyramidal cells [46]. Just like cortical interneurons in the adult brain, CR neurons function as active elements in an early cortical network by integrating synaptic activity of developing pyramidal neurons [46,48].

We found that TC and CR axons concurrently exhibit pronounced growth and pruning, with the balance tipping slightly in favor of growth. TC and CR axons exhibit different modes of elaboration. Structural dynamics were much more pronounced for TC axons than for CR axons. TC axon tips lacked distinct growth cones and grew rapidly in straight paths. In contrast, CR axons had large growth cones and grew slowly along tortuous paths. Branching increased dramatically for TC axons as a function of development. After an early period of branching, CR axons elaborated by growth without branching.

## Results

Here we present our studies on the development of TC and CR axonal arbors in layer 1. We begin by describing long-term in vivo imaging experiments focusing on TC and CR axons. We then analyze the structures and dynamic processes underlying axonal arbor development, including growth cones, axonal trajectories, axonal growth, and pruning. Finally, we investigate how branches are added in TC and CR axons over the first 2 wk of life. Together these observations reveal distinct modes of axonal development for TC and CR axons.

### Imaging Thalamocortical and Cajal-Retzius Axons In Vivo

**Figure 1:**
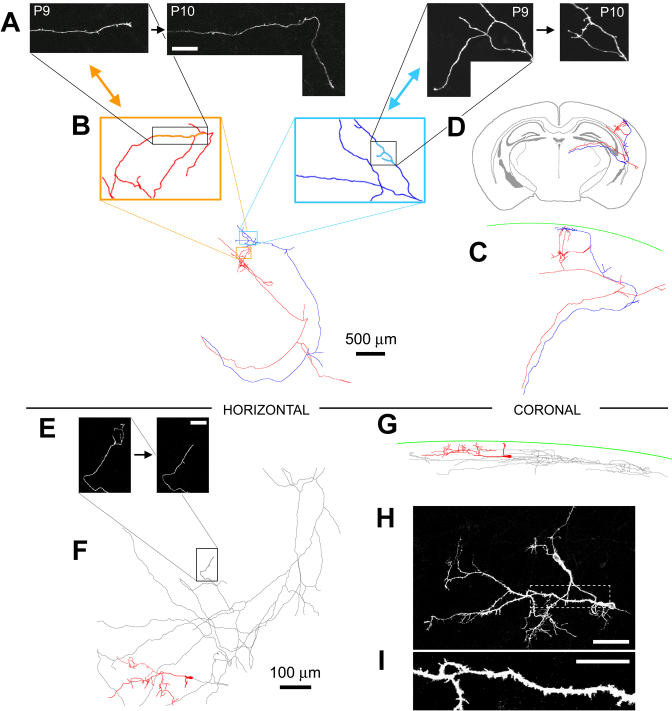
Long-Term In Vivo Two-Photon Imaging of TC and CR Axons in Layer 1 (A and B) Time-lapse images of two different TC axons. (A) The left photomicrographs (corresponding to the orange box in B) are projections of 19 sections; the right (corresponding to the blue box in B) are projections of of 11 sections. Scale bar = 50 μm. (B) Orange and blue boxes contain axons, in horizontal view, reconstructed with BREBIS (see Figure S2E; Materials and Methods) after the last imaging session (P14). The black boxes in (B) correspond to the fields of view of the in vivo images in (A). (C) Axons reconstructed with BREBIS (see Figure S2E; Materials and Methods) in coronal view. The position of the pia is indicated in green. (D) Overlay of the coronal view in (C) on a cartoon of a generic adult mouse brain. (E) Time lapse images of a CR axon (left, 12 sections; right, eight sections). Scale bar = 100 μm. (F and G) Axonal arbor of a CR neuron (gray) imaged in vivo in (E). Views are horizontal (F) and coronal (G). The CR soma and dendrites are drawn in red. (H) Image of the CR dendritic arbor shown in a projection of 14 sections. Note that there are two CR neurons in this image, including the horizontally oriented neuron we traced in red in (F) and (G). Scale bar = 50 μm. (I) High-magnification view of CR dendritic spines (projection of five sections). Scale bar = 25 μm.

We identified a transgenic line (line L21) of mice expressing membrane-targeted GFP in two types of cortical axons [34]. These mice express GFP in a small subset of TC projection neurons from VPm and Pom, both of which project to barrel cortex, as well as in some Cajal-Retzius (CR) cells, the local intracortical interneurons of layer 1 (Figure S1). Pom projections have collaterals in layer 1 [44,45]. A few other neurons expressed GFP during the first 2 wk of postnatal development (e.g., hippocampal pyramidal neurons), but they did not project to somatosensory cortex. Thus, cortical axons within layer 1 presumably belong to either CR cells or thalamocortical projection neurons. GFP expression levels were sufficient for high-contrast in vivo two-photon imaging of individual axons in superficial cortical layers as early as postnatal day 3 (P3), and increased further thereafter (Figure 1).

Axons were identified as CR axons if they could be traced back to the cell body of a CR cell in layer 1 (Figure 1E and 1F). These cells can be recognized on the basis of morphological criteria [48]. Their dendritic trees have spiny branches oriented vertically toward the pia (Figure 1G–1I). Furthermore, CR axons have peculiar branching patterns with sequential bifurcations, offset slightly (5–20 μm) along the dorsoventral axis, with daughter segments running in opposite directions, parallel to the pial surface and always within layer 1 (Figure 1G). In contrast, TC somata were too deep to be imaged in vivo. Instead, the putative TC arbors in layer 1 converged to a single stem that originated deep in the cortex (more than 300 μm below the pia). A subset (*n* = 5) of presumed TC axons were further reconstructed in perfusion-fixed brains using brain reconstruction with en-bloc imaging and slicing (BREBIS) (Figure S2; see Materials and Methods). Such reconstructions revealed that these TC axons coursed through the white matter, consistent with thalamic origin. In some examples (*n* = 3), it was possible to trace these axons all the way to the thalamus (Figure 1A–1D). The morphology of the reconstructed axons, with extensive branching in layers 1 and 5A, matched that of reconstructions of Pom thalamic axons [44].

### Axonal Growth Cones

**Figure 2:**
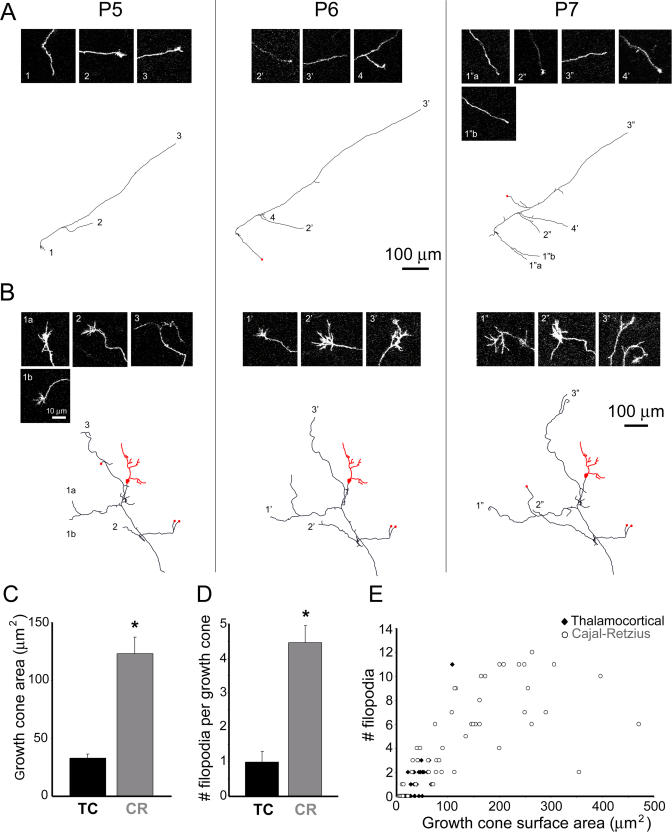
Growth Cone Structure of TC and CR Axons (A) Growth cones at three ages (P5, P6, and P7). Photomicrographs are projections of three or four sections. The numbers in the images correspond to the numbered branch tips in the reconstructions drawn below the images. Red dots mark tips that could not be imaged because their signal was absorbed by overlying blood vessels or bone. Scale bar = 100 μm. (B) Growth cones of CR axons. Images are projections of five to nine sections. The cell body and dendritic tree of the CR neuron are drawn in red. Image numbering, red dots, and scale bars are as in (A). (C) Growth cone surface area for TC and CR axons. For panels C and D we analyzed 38 TC axon tips from five mice and 62 CR axon tips from four mice at ages P5 and P6. Error bars represent SEM. * *p* < 0.01. (D) Number of filopodia per growth cone for TC and CR axons. * *p* < 0.0001. (E) Scatter plot of CR and TC growth cone according to their surface area and number of filopodia.

The complexity of axon growth cones can vary depending on the location of the growing axon [22,23]. Given the identical terrain in which we examined the growth of CR and TC axons, we were surprised to observe that TC and CR axons differed in the structure of their growth cones. The growth cones of TC axons consisted of small swellings that often lacked distinct filopodia (Figure 2A; Video S1). In contrast, during the first week of postnatal development, CR axon tips had large, complex growth cones studded with numerous long filopodia (Figure 2B; Video S2). CR growth cones were four times larger than TC growth cones at P5–P6 (123.0 ± 14.3 μm^2^ versus 32.9 ± 3.2 μm^2^, *p* < 0.01; Figure 2C). Similarly, the number of filopodia per growth cone was higher in CR axons than in TC axons (4.45 ± 0.49 versus 0.97 ± 0.31, *p* < 0.0001; Figure 2D). Growth cones belonging to TC and CR axons were frequently observed in the same region, just a few micrometers apart from one another, suggesting that their structural differences were not imposed by their environment. These differences in growth cone morphology between TC and CR axons persisted throughout the first week of postnatal development. When a branch belonging to either a TC or a CR axon stopped growing (or when it retracted; see below) growth cones could no longer be found at its tip. Thus, the frequency of large growth cones on CR axons decreased with developmental age, and after P9 they were rarely seen. Large growth cones similar to those of CR axons were never encountered in TC axon arbors within superficial cortical layers (Figure 2E).

### Axonal Trajectories

**Figure 3:**
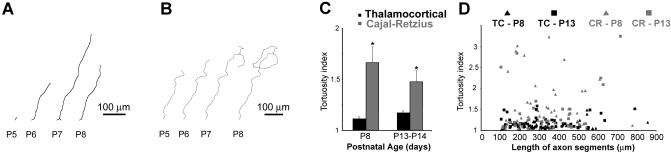
CR and TC Axonal Trajectories (A) Reconstruction of a TC axon segment over time (P5–P8). (B) Reconstruction of a CR axon segment over time (P5–P8). (C) The tortuosity was higher for CR axons than TC axons (P8, * *p* < 0.001, 85 segments; P13–P14, * *p* < 0.0001, 89 segments). Error bars represent SEM. (D) The difference in tortuosity was independent of the length of the analyzed segments.

We analyzed the tortuosity of TC and CR axons. TC axons grew in relatively straight paths (Figure 3A). In contrast, CR growth cones followed tortuous paths (Figure 3B). The large CR growth cones often transiently split into two or more distinct growth cones (Figure S3A). However, these bifurcations were not maintained and instead resulted in sharp turns of the axon trajectory. The differences in tortuosity between TC and CR axons were significant at all ages (Figure 3C and 3D). The tortuosity index was greater for CR cells than TC axons at P8 (1.67 ± 0.2 versus 1.11 ± 0.02, *p* < 0.001), and at P13 (1.48 ± 0.1 versus 1.2 ± 0.02, *p* < 0.0001). The tortuosity of TC axons increased somewhat with age due to the late addition of short twisted branches. Note that even those late branches lacked large growth cones, implying that the difference between CR and TC axon growth cones was not due to the developmental (or chronological) age of the arbors. The disparity between TC and CR axonal trajectories suggests differences in the mechanisms of guidance for these types of neuron subtypes. In particular, the tortuous paths of CR axons suggest intimate interactions with local guideposts and specificity at the level of axon trajectory [52].

### Axonal Growth and Retraction

**Figure 4:**
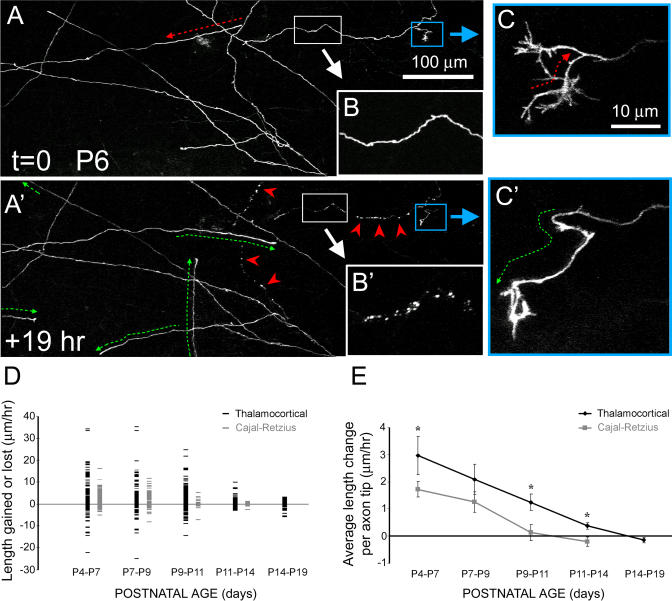
Axon Growth and Pruning (A and A′) Representative axon tips imaged at two time points 19 h apart (images are projections of 12–20 sections). Some TC axon tips grew (green arrows), while others retracted (red arrows), sometimes only to grow again in a slightly different direction. Pruning of some TC axons involved fragmentation of the local arbor (red arrowheads in A′). CR axons could also be seen in the same regions of interest (blue inset). (B and B′) Higher-magnification view of TC axonal degeneration (projections of 11 and 18 sections). For earlier stages of degeneration see Figure S4B. (C and C′) Higher-magnification view of the CR axon growth cones. (D) Plot of growth and retraction at individual axon tips throughout postnatal development. Each line represents a single axon tip (1,036 TC axon tips and 292 CR axon tips). Imaging sessions were ∼10–30 h apart and the length changes are given as rates. The rates therefore represent a lower estimate of the actual movement dynamics. (E) Average length change at individual axon tips, including growth and retraction. The average growth at TC axon tips was always greater than at CR axon tips. See also Figure S4C. Error bars represent SEM. * *p* < 0.05.

Does pruning of cortical axons follow a period of overgrowth [12,53]? We investigated whether local pruning occurs in vivo within the axon's target region, and if so, whether it occurs concurrently with or subsequent to a period of growth (Figure 4). We also asked whether rates of growth are different in CR versus TC axons, as might have been predicted by their dissimilar growth cone morphologies [21–23]. TC and CR axon tips were imaged 10–30 h over a range of developmental ages (P4–P19). Some tips grew over considerable distances between imaging sessions, while others retracted (Figure 4A; see also Figure S4A and S4B). Individual axonal arbors simultaneously exhibited growth and retraction at different branch tips. There were no discernable morphological clues that helped predict whether a tip would grow or retract. TC axon tips grew or retracted at high rates, up to 35 μm/h. Length changes were less rapid for CR axon tips (Figure 4C). The fraction of axon tips growing (65%) and retracting (35%) was similar for CR and TC, and across different ages (unpublished data). In the first week of postnatal development, rapid growth or retraction occurred at most branch tips, but after P12, axon tips grew or pulled back only over short distances (Figure 4D; see also Figure S4C). On average, growth was almost balanced by retraction, although the net balance tipped in favor of growth until the third week of postnatal development (Figure 4E). The overall net growth of axon tips was higher for TC axons than CR axons at every time point examined.

### Two Types of Pruning: Axonal Degeneration as Part of Normal Development

**Figure 5:**
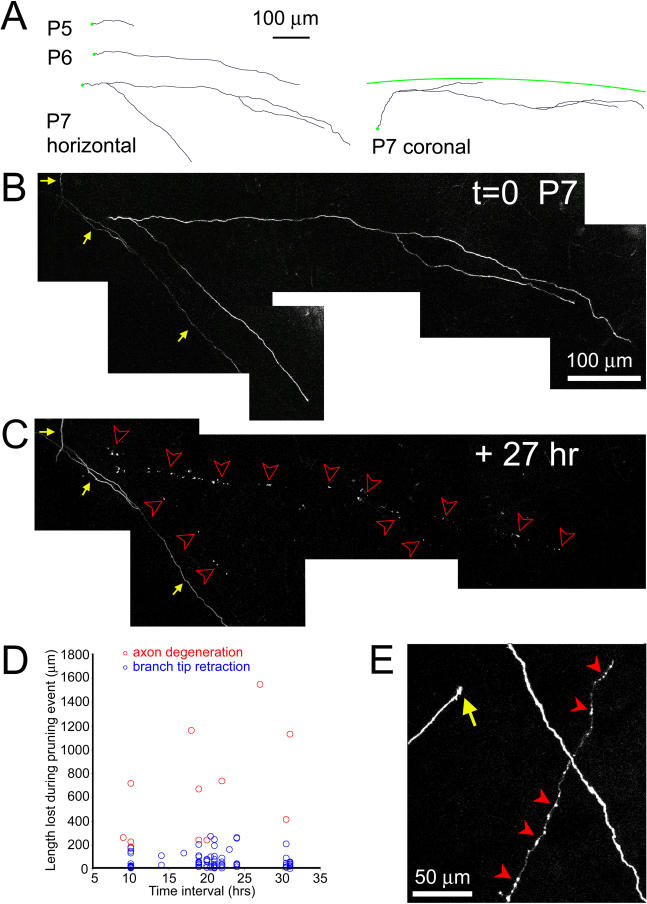
Large Portions of TC Axon Arbors Are Pruned by Axonal Degeneration (A) Horizontal views (left) of tracings of a TC axon imaged in vivo from P5 to P7. The coronal view of the P7 trace (right) shows that branches with layers 1 and 2/3 converged upon a single parent axon stem arising from deep in cortex. The pia is drawn in green. (B) In vivo image of the same axon as in (A) at P7 (projections of 10–22 sections). A neighboring axon (yellow arrows) can also be seen. (C) Same axons as in (A) 27 h later. The large axon arbor has degenerated, and fragmented debris (red arrow heads) is found in its place. (D) Quantification of length lost through branch tip retraction (blue) and axon degeneration (red) for *n* = 92 pruning events. When adjusted for a 24-h interval, the average length lost per pruning event at P5–P9 was 782.3 ± 134.7 μm for degenerating arbors versus 80.6 ± 10.1 μm for retracting segments (*p* < 0.0001). (E) Axon degeneration in a control, in vivo imaging-naïve brain at P4 (projection of 38 sections). The animal was perfused transcardially with cold 4% paraformaldehyde and 10% sucrose in PBS, in order to avoid rosary-type beading that is commonly seen in perfusion-fixed preparations of rodent brains at these young ages. Two intact axons can be seen coursing through the neuropil, one of which has a small growth cone (yellow arrow) typical of TC axons. A third axon is seen undergoing degeneration (red arrowheads), the fragmented debris appearing indistinguishable from the fragmented axons encountered in vivo (compare to Figures 4A′, 5B, and S4B).

Pruning is one of the cardinal features of axon arbor development. We observed two types of axonal pruning. In addition to branch tip retractions, pruning also occurred by degeneration of large portions of axonal arbors (Figures 4 and 5). Degeneration began with beading and swelling of the axon shaft, followed by detachment of branches from their parent shafts (Figure S4B). The degenerated axon shafts then disintegrated into small fragments (Figure 5C), the entire process taking less than 12 h. The debris then disappeared within 24–48 h, presumably after being cleared by glia. This differed from retraction in that retracting axons did not leave fluorescent debris behind (Figure S4D). It was not possible to predict degeneration based on axonal morphology. Axonal degeneration often spanned all the imaged axon arbor of one neuron (Figure 5). However, degeneration was typically restricted to a single axon in the field of view, as opposed to retraction, which affected branch tips of multiple neighboring axons. This suggests that axonal degeneration is intrinsic to a given axon and not a response of a population of axons to an extrinsic signal. To rule out the possibility that axonal degeneration was a pathologic phenomenon related to the surgery or in vivo imaging, we also imaged brains of perfusion-fixed, naive mice. In these control brains, axonal degeneration was also detected at the same young ages at a similar frequency (Figure 5E), implying that degeneration is a normal developmental process.

Axon degeneration was relatively infrequent (affecting less than 5% of GFP-positive axons). Degeneration was observed only at younger ages (P4–P9), and in TC axons but not in CR axons. Axon degeneration typically involved long stretches of TC axon with multiple branches. Sometimes an entire TC arbor that had grown over several days in layer 1 suddenly degenerated all the way down to deep cortical layers as far as we could image (Figure 5A). Axon segments that underwent degeneration were always much larger than those undergoing retraction. The average size was 782.3 ± 134.7 μm for degenerating arbors versus 80.6 ± 10.1 μm for retracting segments at P5–P9 (Figure 5D; *p* < 0.0001). Since in many cases the entire axon in the field of view was fragmented, our estimates of the average length lost through axonal degeneration represent a lower bound. Therefore, degeneration constitutes a mechanism of axon elimination that is distinct from retraction and operates over longer lengths of axon.

### Axonal Branching

Branch formation has been proposed to occur via one of three distinct mechanisms: Splitting of growth cones, delayed growth cone branching, and interstitial (growth cone-independent) branching [18,22,24]. How do neocortical axons branch in vivo?

**Figure 6:**
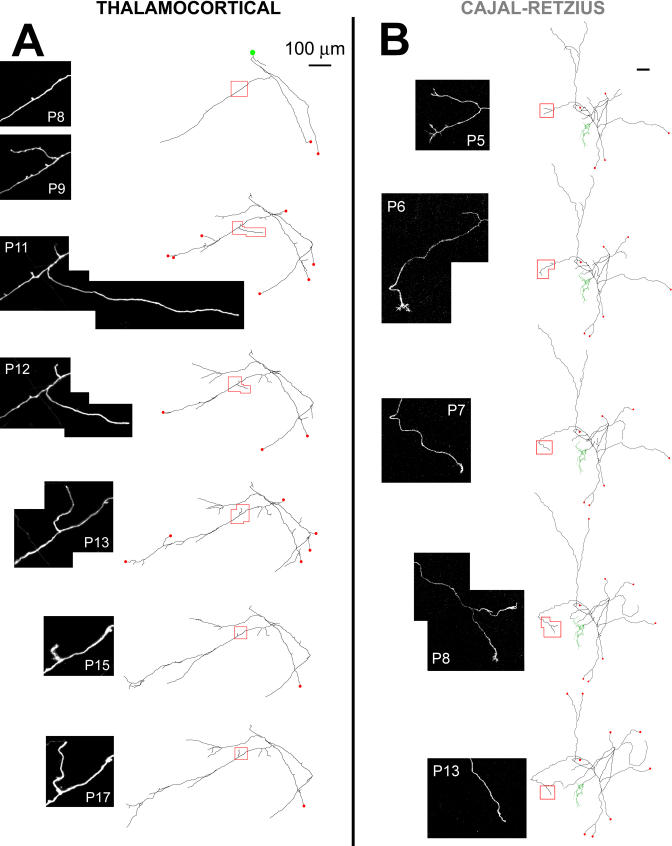
Dynamics of Branch Additions and Retractions (A) Reconstruction of a representative TC axon imaged from P8 to P17. Insets show photomicrographs corresponding to regions of interest indicated by red boxes on drawings to the right. See also Figure S3B. Images are projections of 8–19 sections. Red dots mark tips that could not be imaged. Note that the tracing corresponding to the P9 image is not shown. (B) Reconstruction of a representative CR axon imaged between P5 and P13. Image insets are projections of 13–27 sections. The CR soma and dendritic tree are drawn in green.

We observed interstitial branch formation in TC and CR axons (Figure 6). In TC axons, small growth cones appeared de novo from the main shaft (see Figure S3B), and then elongated orthogonal to the parent axon. In the case of CR axons, the new growth cones also emerged from the parent shaft (unpublished data), and then quickly enlarged to assume their typical large configuration. CR axon growth cones (but not TC axons) often split into two or more segments, but never produced two permanent daughter branches (see Figure S3A). These observations imply that, during axon elaboration within the target region, interstitial branching is the dominant form of axonal branching in the neocortex.

**Figure 7:**
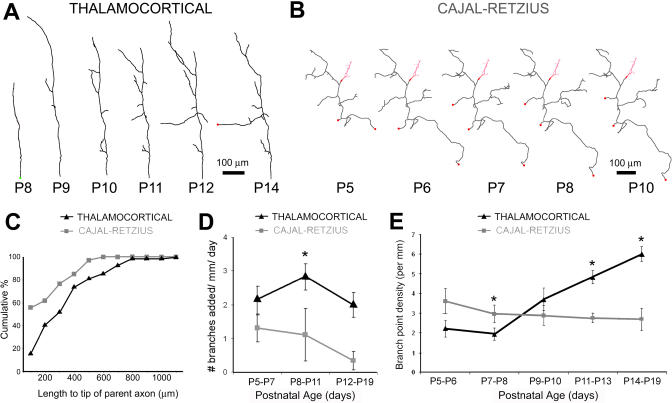
Development of Axonal Complexity (A) Tracings of a representative TC axon from P8 to P14. (B) Tracings of a representative CR axon from P5 to P10. (C) Location of branch additions with respect to tip of parent axon for 69 new TC branches and 34 new CR branches. The difference between TC and CR axons was significant (*p* < 0.05). (D) The rate of branch additions per millimeter of axon per day was greater in TC axons (*n* = 30) than in CR axons (*n* = 12). Error bars represent SEM. * The difference was significant at P8–P11 (*p* < 0.05). (E) Density of branch points versus postnatal age. The branch point density gradually increases for TC axons (*n* = 219) between P5–P6 and P14–P19 (*p* < 0.0001), whereas it decreases slightly for CR axons (*n* = 76) during the same time period (*p* = 0.4). The overall difference in these trends between TC and CR axons was significant (*p* < 0.05). Error bars represent SEM. * The density of branch points for TC axons, which was lower than that of CR axons at P5–P6 (*p* = 0.064) and at P7–P8 (*p* = 0.05), was later significantly higher at P11–P13 (*p* < 0.01) and at P14–P19 (*p* < 0.001).

However, the placement of new branches within the developing arbor differed between TC and CR axons. Interstitial branching in TC axons occurred essentially uniformly along the principal axon segments (Figures 6A and 7A). In contrast, new branches in CR axons appeared closely behind the leading growth cone (Figures 6B and 7B). Out of 69 new branches added to TC axons, only 11 (16%) appeared within 100 μm of the tip, compared to 19 (56%) out of 34 new branches added to CR axons in that location (*p* < 0.005; Figure 7C).

Our ability to image the same axons over periods of up to 2 wk allowed us to determine the developmental progression by which axonal arbors elaborate. These long-term imaging studies revealed profound differences between TC and CR axons. During the first week of postnatal development, many TC axons were initially composed of very few (1–3) long branches that extended in straight paths for hundreds of microns in layer 1, without secondary branches (Figure 7A; see also Figures 2A and 6A). Over the next few days, short branches appeared all along these parent axons at increasing rates (from 2.2 ± 0.5 branches per millimeter of axon per day at P5–P7 to 2.8 ± 0.4 at P8–P11; *p* = 0.09; Figure 7D). Many of these early branches were unstable with short lifetimes, but during the second week of postnatal development, new branches became stabilized. Thus, between P5–P6 and P14–P19 the branch density (number of branch points per millimeter of axon) increased by a factor of three (Figure 7E; from 2.2 ± 0.4 to 6.0 ± 0.4 branch points per millimeter of axon; *p* < 0.0001).

At the time of the first imaging session, around P5, CR axons already had numerous (more than ten) branches close to the soma, and most branch tips were still growing (Figure 7B; see also Figures 2B and 6B). During the first postnatal week, branches were added at a low rate (1.3 branches per millimeter of axon per day), which continued to decrease with development (Figure 7D). The rate of branch addition for CR axons across all ages was significantly lower than for TC axons (*p* < 0.05). Because the main form of growth for CR axons was extension at axon tips without branching, the branch density decreased between P5–P6 and P14–P19 from 3.6 ± 0.6 branch points per millimeter at P5–P6 to 2.7 ± 0.6 at P14–P19 (*p* = 0.4). The difference in branch density trends during postnatal development between TC and CR axons was significant (*p* < 0.05). During the third week of postnatal life, TC axons changed little, whereas CR branch tips retracted slightly. CR neurons and their axons became dimmer, presumably because of decreased GFP expression, and eventually disappeared.

## Discussion

The development of axonal arbors is a critical step in the establishment of precise neural circuits. To discover the modes of axonal elaboration, we imaged axons of TC projection neurons and CR interneurons from early postnatal days until their arbors were mature, in the third week of postnatal development. These axons were intermingled within layer 1 and therefore experienced identical extracellular influences. We found that the elaboration of both CR and TC axons relies on a fine balance of growth and pruning. TC and CR axons have distinct structures and elaborate with different dynamics, suggesting that different neuronal subtypes use different intrinsic developmental programs to innervate their target cells. In addition, our data are relevant to controversies regarding the modes of axon pruning and branching.

### Axonal Trajectory, Growth, and Pruning

Within cortical layer 1, TC axons have small growth cones and follow essentially straight paths, while CR axons have large growth cones that advance in tortuous paths. Elaborate growth cones are thought to be necessary for contact-mediated guidance [22]. Since tortuous axonal paths may imply interactions between axon growth cones and local guidance cues [52], our findings imply that TC axons might explore the local environment with little guidance from local cues, whereas CR axons are strongly shaped by such signals.

Axon growth and pruning are cardinal features of nervous system development. Based on observations from fixed mammalian axons [4–7], it is often assumed that early overgrowth is followed by pruning [54]. But concurrent growth and pruning have also been described, especially in lower vertebrates such as *Xenopus* tadpoles [10,14,15]. We studied growth and pruning within the target area of mammalian cortical axons in quantitative detail and find that branch tip growth and retraction events occurred simultaneously. Growth and pruning were almost balanced, with a small preference toward growth (see Figure 4E). As a result, the total size of the axonal arbor increased monotonically with age until the third postnatal week.

We observed two distinct forms of axonal pruning: branch tip retraction and degeneration. Branch tip retraction occurred in both TC and CR axons. Relatively small branch segments (tens of micrometers) were retracted, leaving behind no detectable debris (see Figure 4). Axonal degeneration was seen only in TC axonal arbors and resulted in the elimination of large portions (hundreds of micrometers) of the axonal arbor (see Figure 5). Degeneration did not proceed sequentially in one direction (e.g., distal-to-proximal) but instead affected the entire axon synchronously. This is similar to what happens during Wallerian degeneration in transected nerves, where large-scale fragmentation of the entire disconnected axon (several hundred microns in length) occurs suddenly, within a few minutes [30]. The process of degeneration in TC axons occurred through beading and then fragmentation, which is also reminiscent of Wallerian degeneration. Similar forms of pruning have been observed in the *Drosophila* mushroom body [28] and in cultured hippocampal axons [32]. In our experiments, axon degeneration was not likely a pathologic phenomenon related to imaging. First, degenerating axons were seen only during a specific time period (P4–P9), suggesting that it is a developmentally regulated process. Second, axonal degeneration occurred at various times after the window surgery (up to 3 d later). Third, only a minority (less than 5%) of axons were affected by this type of pruning. Finally, fragmented axons were also identified in control, unoperated, perfusion-fixed mice at equivalent ages (see Figure 5E). Degeneration of TC axons is also unlikely to be due to apoptosis of neurons in thalamus. The wave of naturally occurring cell death in the thalamus occurs mostly before birth in the mouse [55–57]. Furthermore, inhibition of caspase activity does not prevent Wallerian degeneration or pruning of *Drosophila* mushroom body axons [28,58].

Both axon degeneration and branch tip retraction in cortical axons differed from axosome shedding at the neuromuscular junction [59]. Axosome shedding involves the loss of membranous organelles as axons slowly retract. In contrast, axonal degeneration occurred essentially synchronously along long segments of axons. Axon retraction did not involve retraction bulbs (seen in axosome shedding), and fragmented debris was not observed, even when we imaged at frequent intervals (Figure S4D). Axosome shedding may, therefore, represent a distinct third mechanism of pruning in the peripheral nervous system. During development, axonal retraction and degeneration probably play different roles in wiring the neocortex. Retraction is likely involved in the local refinement within a target area, while degeneration operates at the level of projections. It will be interesting to determine if axonal degeneration in developing mouse TC axons and *Drosophila* mushroom body neurons are mechanistically related. Furthermore, it will also be important to explore the mechanisms that govern maintenance versus retraction of branches in TC and CR axons, including the roles of neuronal activity and synaptogenesis.

### Axonal Branching

Experiments in the spinal cord and neocortex have demonstrated that many axons can branch at interstitial sites, anywhere along the parent axon shaft [27,60]. However, some controversy remains regarding the role of the growth cones in branching [18], as some axons seem to exploit the splitting of growth cones for branching [25]. Growth cones of CR axons often split transiently into two branches, but one of these branches was subsequently retracted. Splitting growth cones therefore never gave rise to two bona fide daughter branches. Rather, time lapse imaging of individual CR growth cones suggests that growth cone splitting may be involved in guidance/turning behaviors (see Figure S3A). Our data show that the vast majority of branches are formed at interstitial sites (see Figure 6), inconsistent with a direct role for growth cone splitting in branch formation.

Axon growth cones have also been implicated in interstitial branching [24]. Two types of interstitial branching have been described. Experiments in dissociated cultures of cortical neurons have shown that growth cones can leave behind sites of unstable cytoskeleton, which later become branch points through a process known as delayed interstitial branching [26]. In other cases, interstitial branches appear days after the main axon stopped growing [24]. We found that TC axons add new branches all along their arbor, inconsistent with delayed interstitial branching. In contrast, CR axons preferentially add branches close to the growing tips, suggesting that a process similar to delayed interstitial branching may occur in CR neurons in vivo. However, we did not observe filopodia or lamellar remnants being left behind by the advancing growth cone, as described previously for delayed interstitial branching [26].

### Distinct Modes of Elaboration for TC and CR Axons

The function of cortical circuits in the mature brain relies on a precise circuit diagram. The physical organization of axonal and dendritic arbors is the structural basis of functional circuits. The elaboration of axons involves both intrinsic programs and extrinsic factors, including guidance cues and neural activity [10,11,61]. Because axons of different cell types are molecularly distinct, they are likely to respond differently to such external cues and grow in different ways. Time-lapse imaging revealed that TC and CR axons develop with distinct strategies over the first 2 wk of development (Figure S5; Videos S3 and S4). TC axons grow and retract rapidly, following straight paths. With developmental age they become more complex, adding numerous branches to their arbors. CR axons grow and retract more slowly. Their elaboration in the postnatal brain is primarily through elongation of tortuous branch tips, with negligible interstitial branching.

Some of these differences in behavior between CR and TC axons were due to differences in their developmental time-course. For example, much of CR branching had occurred before our first imaging session, while most of the TC branching within layer 1 was yet to occur. Yet, CR and TC neurons are born at approximately the same embryonic age [38,46], and, at the ages we studied, CR axons still had many large growth cones and grew long distances over the imaging period. For these reasons, the differences in growth cone structure, axonal trajectory, and growth rates between these two cell types cannot be attributed to differences in developmental maturity between CR and TC axons.

Axonal structure is cell-type dependent. For example, cortical pyramidal cell axons have straight paths, while cortical inhibitory interneuron axons have tortuous paths [62,63]. Our data show that this distinction holds for TC projection neuron and CR interneuron axons. Computational analysis has further shown that projection neuron axons make as many “contacts” with synaptically unconnected dendrites as with connected dendrites [52,64]. In contrast, interneurons make more contacts with their postsynaptic partners compared to other neurons in the vicinity. The different modes of elaboration we observed for TC and CR axons are consistent with this view. TC axons elaborate with large-scale growth and retraction, in straight paths, suggesting that these axons may be stabilized by activity-dependent selection [10]. In contrast, CR axons elaborate more gradually in tortuous paths, suggesting that these axons are strongly influenced by extracellular guidance cues.

### Axon Elaboration and Critical Periods

Both the addition of new branches (Figure 7D) and the degree of growth and retraction at individual tips of TC axons (see Figure 4D) diminished after the second postnatal week, suggesting that arbors reach a mature state around that time. Interestingly, the end of the second postnatal week coincides with the end of the critical period for barrel cortex. During this critical period, layer 2/3 sensory maps [65,66] and circuits [67] display rapid developmental and experience-based plasticity. Moreover, the maturation of dendritic branching [68] and spine motility [65] of layer 2/3 pyramidal neurons are also affected by sensory deprivation. It is likely that local axonal growth and pruning contribute to experience-dependent development of cortical circuits. Indeed, activity-dependent rearrangements of thalamocortical axon collaterals have been demonstrated in vitro [11,69]. It remains to be seen whether manipulations of sensory experience, for example by whisker trimming, similarly affect axonal development.

## Materials and Methods

### Animals and surgery

All experimental protocols were conducted according to the National Institutes of Health guidelines for animal research and were approved by the Institutional Animal Care and Use Committee at Cold Spring Harbor Laboratory. We used male and female c57/Bl6 transgenic mice in which a *thy1* promoter drove the expression of GFP that was targeted to the cell membrane via a palmytoylated site (mGFP, line L21 [34]). Throughout the first 2 wk of postnatal development, these mice express GFP in Cajal-Retzius neurons, thalamic neurons in the Pom and VPm nuclei, and in CA1 pyramidal neurons in hippocampus. Importantly, cortical pyramidal neurons begin to express GFP only toward the end of the second postnatal week (with the exception of a small number of pyramidal neurons in the cingulate cortex). At P3–P12, mice were deeply anesthetized with an isoflurane-oxygen mixture delivered by an anesthesia regulator (SurgiVet, Waukesha, Wisconsin, United States) or with an intraperitoneal injection of a mixture of ketamine (0.13 mg/g body weight) and xylazine (0.01 mg/g). Following a midline scalp incision, the skull overlying the right somatosensory and visual cortices was carefully removed, leaving the dura intact. The dura was covered by a thin layer of 1.2% low-melting-point agarose (Type IIIA, Sigma, St. Louis, Missouri, United States) dissolved in Hepes-buffered artificial cerebrospinal fluid, and then a custom-made round cover glass (No. 1) was quickly laid over the agarose. This “window” (see Figure S2A) was sealed in place with dental cement. A titanium bar was also attached to the skull to later secure the mouse to the stage of the microscope. Between imaging sessions mice were housed with littermates and their mothers. The surgery itself seemed innocuous to the mice, as they recovered nicely from the surgeries and anesthesia, gained weight, and behaved indistinguishably from their unoperated littermates.

### Imaging

Imaging began after a recovery period of at least 1 h. Animals were anesthetized with an isoflurane-oxygen mixture. The animals woke up within a few minutes after stopping the flow of anesthetic. In vivo images of GFP-expressing neurons were acquired with a custom-built two-photon microscope [65], using a Ti:sapphire laser (Tsunami, Spectra Physics, Mountain View, California, United States), running at approximately 910 nm, pumped by a 10 W solid state laser (Millenia X, Spectra Physics). The objective (40×, 0.8 NA) and scan lens were from Zeiss (Oberkochen, Germany), the trinoc from Olympus (Tokyo, Japan), and the photomultiplier tube from Hamamatsu (Hamamatsu City, Japan). Detection optics with large apertures provided optimal fluorescence detection [70]. Image acquisition was achieved with ScanImage software custom-written in MatLab (MathWorks, Natick, Massachusetts, United States) [71]. Slightly overlapping image stacks (up to 40 per animal) were taken at low zoom (approximately 210 × 230 μm field of view; see Figure S2D). For high-magnification imaging of axon growth cone dynamics smaller ROIs (approximately 50 × 50 μm) were selected at random or centered on axon tips. All image stacks consisted of optical sections with 512 × 512 pixels separated by 1- to 3-μm steps. By following axons over long distances, we could distinguish between axons belonging to CR neurons (identified by their location within layer 1 and their characteristic dendritic trees; see Figure 1G and 1H) and TC axons that originated from below layer 2/3 (> 300 μm). Care was taken to achieve close to identical fluorescence levels across imaged regions and imaging sessions by adjusting the laser intensity at deeper focal planes or by compensating for increased expression levels of GFP or darkening of a particular ROI due to growing blood vessels or skull bone. Imaged axons were within 450 μm from the surface of the brain. All images in the Figures are projections of three-dimensional stacks. Labeling in the line 21 mGFP mice was very sparse (see Figure S2D), especially in mice less than 1 wk of age; this facilitated the task of tracing individual axons. In some time-lapse experiments, dendrites and axons expressing GFP accumulated at older ages. Therefore, in some Figures, particularly those consisting of projections of more than ten sections, distracting fluorescent processes were digitally removed for presentation.

### BREBIS

Retrospective reconstructions of selected axonal arbors were used to confirm their identity as TC axons. After the last imaging sessions, mice were perfused transcardially with 4% paraformaldehyde, and their brains were carefully removed and blocked to remove the cerebellum and left hemisphere. Brains were post-fixed overnight in the same fixative and then immersed in low-melting-point agarose and mounted on the well of a vibratome slicer (Leica) that was also fitted to be mounted on the stage of the two-photon microscope. Previously imaged axons were once again identified using blood vessel imprints on the dura (see Figure S2C), or by using injections of fluorescent beads as fiducial points. These axons were imaged in the same orientation as during in vivo imaging and as deep as possible (often up to 800 μm in a single set of image stacks). Next, the most superficial 300–600 μm of brain were sliced off and the brain was re-imaged, always keeping track of the location of the imaged axon. Following several cycles of serial imaging and slicing (see Figure S2E), the axon could be traced from layer 1 all the way down to the thalamus (see Figure 1). Naïve animals were also processed for BREBIS and analyzed as controls (*n* = 3). These axons were imaged under identical conditions to in vivo imaging (optics, magnification, orientation) in whole mounts of the fixed brains. We randomly selected axon segments of similar TC axons imaged in vivo and confirmed with BREBIS that they indeed originated from the white matter or from thalamus. Since it was not possible to reconstruct every axon that was imaged, it is possible that some putative TC axons originated outside the thalamus, for example in the brainstem. However, such brainstem projections to neocortex, including monoaminergic axons, innervate cortex in a diffuse pattern that spans many layers, and are inconsistent with those we observed for putative TC axons [72].

### Tracing of axons and analysis

The data in this study come from 22 mice imaged in vivo. Nine mice were imaged over multiple days, and 13 mice were imaged over two time-points at least one day apart. In addition, six control mice were imaged only after fixation. The data analyzed for this study were collected over a total of more than 100 separate imaging sessions. We analyzed a total of 253 mm of TC axons and 99 mm of CR axons. Tracing and analysis were performed independently by two investigators (CPC and RW). Axons were traced in three dimensions within tiled stacks using the confocal module of Neurolucida (Microbrightfield, Williston, Vermont, United States; see Figure 2D). From these tracings we could easily derive data regarding axon segment lengths (growth and retraction over time), branch densities, and axon tortuosity. Axon extensions were considered to be true branches (as opposed to filopodia or elongated terminal boutons) if they measured at least 7.5 μm in length. We also imaged fixed brains that had never undergone surgery or in vivo imaging (*n* = 3 mice, nine axons, measuring a total of 22 mm) at P7–P8 and at P14–P15 to study the effects of surgery and/or chronic imaging on axonal development. The density of branch points was not different from that of experimental mice (at P7–P8, 2.1 ± 0.5 branch points per millimeter of axon in controls versus 1.9 ± 0.3 in experimental mice, *p* = 0.88; and at P14–P15, 5.4 ± 0.6 branch points per millimeter of axon in controls versus 5.9 ± 0.6 in experimental mice, *p* = 0.63; Figure S6), suggesting that there were no deleterious effects due to the surgery or chronic imaging.

For the growth cone comparison (see Figure 2), we analyzed 62 CR growth cones from four mice and 38 TC growth cones from five mice at ages P5 and P6. Growth cone size was determined by tracing a contour encompassing the base of the growth cone and the tips of all filopodia. For bulbous growth cones lacking filopodia, a contour of the bulbous swelling was measured. For tortuosity data (see Figure 3), we analyzed 93 TC axon segments from six mice and 81 CR axon segments from six mice, and the average length of those segments was 328 μm and 332 μm (*p* > 0.34), respectively. Only axons from mice that had been imaged both at P8 and at P13/P14 were included in the tortuosity analysis. Axon arbors were first broken down into the individual branches and then the longest segments were further broken down into smaller segments of approximately similar sizes such that all tortuosity data was were obtained from axon segments measuring more than 100 μm and less than 850 μm. The tortuosity index is the ratio of the true contour length of the axon segment divided by the end-to-end distance. The number of mice/axons/tips analyzed for short-term growth and retraction (see Figure 4) was as follows: for TC axons: at P4–P7, 9/46/128; at P7–P9, 8/33/145; at P9–P11, 5/32/240; at P11–P14, 5/32/363; and at P14–P19, 3/21/160. For CR axons, values were: at P4–P7, 8/43/137; at P7–P9, 7/29/92; at P9–P11, 3/11/28; and at P11–P14, 4/10/35. For those data, in animals older than P10 we analyzed only axons that had been already followed for several days since P8 or younger. This was a precaution to avoid collecting data from axons belonging to cortical pyramidal neurons, which begin to express GFP toward the end of the second postnatal week. For the comparison between axon degeneration and retraction (see Figure 5), 13 degenerating arbors and 79 retracting segments were analyzed. Branch density data were obtained only for axon segments larger than 100 μm. The number of mice/axons analyzed for branch density measurements were as follows: for TC axons: at P5–P7, 7/59; at P7–P8, 9/52; at P9–P10, 4/33; at P11–P13, 6/48; at P14–P19, 5/27. For CR axons, values were: at P5–P7, 4/29; at P7–P8, 4/29; at P9–P10, 2/7; at P11–P14, 3/11. Finally, for measurements of branch additions we analyzed 30 TC axons from eight mice and 12 CR axons from six mice. Significance was set at *p* < 0.05 and evaluated using Student's *t*-test. Error bars represent the standard error of the mean (SEM).

## Supporting Information

Figure S1 GFP Expression Pattern in L21 Transgenic Mice Immunocytochemistry was performed to detect GFP on coronal sections from line L21 brains at P7.
(A) Primary somatosensory cortex. Note that cortical pyramidal neurons do not express GFP at this early stage. Branching of TC axons in layer 4 delineates barrels (asterisks). Scale bar = 100 μm.
(B) High magnification of inset in (A) showing axons in layer 1 (arrows). Scale bar = 10 μm.
(C) Coronal view of a CR cell (arrowhead) and CR axon (arrow) in layer 1. Scale bar = 60 μm.
(D) Thalamus (Th). Insets 1 and 2 are shown at higher magnification in E and F, respectively. Scale bar = 220 μm.
(E and F) Higher-magnification views of the thalamic neurons shown in (D). Cell bodies of bright relay neurons are indicated by arrow heads. Fi, fimbria of the hippocampus. Scale bars = 35 μm.
(G) Primary visual cortex and dorsal hippocampus. Insets are shown at higher magnification in H and I, respectively. Scale bar = 200 μm.
(H and I) Higher-magnification views of TC axons (arrows) in layers 1 (H) and 2/3 (I) of visual cortex shown in (G). Scale bars = 60 μm (H) and 30 μm (I).
(6.3 MB TIF).

Figure S2 Methods To image developing axons in vivo, we performed window surgeries on transgenic mice expressing membrane-bound GFP under the *thy1* promoter.
(A) Photograph of a P3 mouse shortly after surgery. A titanium bar was used to immobilize the animal onto the microscope stage.
(B) Axons were imaged in superficial layers of cortex over several days and up to 2 wk.
(C) The unique pattern of blood vessels in each mouse was used to find the regions of interest from one imaging session to the next.
(D) Axons were then traced from overlapping stacks of images.
(E) In order to confirm the identity of thalamocortical axons, some animals were fixed by perfusion and the previously imaged axons were reconstructed using BREBIS (see Figure 1; Materials and Methods)
(3.3 MB PDF).

Figure S3 Tortuous Path of CR Axon Growth Cone and Interstitial Branching in TC Axon (A) Time lapse sequence showing the tortuous path of a CR axon growth cone. Images are projections of 13–19 sections.
(B) Time lapse sequence of the birth of a TC axon branch, starting with the de novo appearance of an interstitial growth cone within the shaft of the TC axon. Time stamps in hours.
(3.1 MB PDF).

Figure S4 Examples of Growth, Branch Retraction, and Axonal Degeneration (A and B) Additional examples of growth (green arrows) and retraction (red arrows) of TC axon tips (see also Figure 4). (A) Initial imaging session; (B) Same field of view imaged 10 h later. The fragmented axon shown here (red arrow heads) is at an earlier stage of degeneration than the one shown in Figures 3 and 4.
(C) Average length gained (growth) or lost (retraction only) for TC (black) and CR (gray) axon tips at various ages throughout postnatal development. The differences in growth between TC and CR axons were statistically significant at P4–P7 (*p* < 0.0001), P9–P11 (*p* < 0.05), and P11–P13 (*p* < 0.05). The differences in retraction between TC and CR axons were statistically significant at P4–P7 (*p* < 0.0001), P7–P9 (*p* < 0.001), and P9–P11 (*p* < 0.01).
(D) Time lapse images obtained at 1-h intervals of a cortical axon from a P4 mouse. Red arrows indicate branches that are retracted. Note that branch tip retraction is distinct from axosomal shedding in that it does not involve retraction bulbs and does not leave a trail of debris. Scale bar = 5 μm. Error bars represent the SEM.
(2.0 MB PDF).

Figure S5 Summary Cartoon of the Different Modes of Axon Elaboration Used by Local Intracortical Axons (CR) and Long-Range Extracortical Axons (TC) (A) TC axons, which lack exuberant growth cones, grow fast during the first postnatal week. But this remarkable growth is accompanied by a surprisingly high amount of local pruning. At later stages, many branches are added to the original “bare” skeleton, and although growth slows down, so does the amount of branch retraction.
(B) CR axons branch extensively during the first week of postnatal development. Their large growth cones guide the axon tips in a slow, twisting path, with little pruning. Later, growth cones disappear and growth and branching slow down. Some branches are eventually lost.
Thus, two modes of axon arbor elaboration exist in early postnatal cortex. On the one hand, local interneurons grow slowly in a meandering course, making few mistakes. On the other hand, long-range projection axons grow fast, leading to frequent errors and backtracking. Given the identical terrain upon which these axons grow, the differences must reflect intrinsic differences in the axons themselves, such as in their ability to sense gradients of signaling molecules or in their method of synapse formation.
(265 KB PDF).

Figure S6 Density of Branch Points in Imaged Mice Matches That in Control Mice To control for possible deleterious effects of chronic imaging, we examined perfusion-fixed, in vivo imaging-naïve mice (*n* = 3). The density of branch points in these control mice was not different than that of imaged mice at P8 (*p* = 0.88) and P14–P15 (*p* = 0.63). Time stamps in hours. Error bars represent the SEM.
(194 KB PDF).

Video S1 TC Growth Cone The time stamp is given in minutes.
(1.1 MB AVI).

Video S2 CR Growth Cone The time stamp is given in minutes.
(1.9 MB AVI).

Video S3 TC Axon Cartoon (2.3 MB AVI).

Video S4 CR Axon Cartoon (2.2 MB AVI).

## Abbreviations

BREBIS: brain reconstruction with en-bloc imaging and slicing
CR: Cajal-Retzius
E[number]: embryonic day [number]
GFP: green fluorescent protein
P[number]: postnatal day [number]
Pom: medial part of the posterior nucleus of the thalamus
SEM: standard error of the mean
TC: thalamocortical
VPm: ventral posteromedial nucleus of the thalamus
