# Supplementary material for: Diverse Modes of Axon Elaboration in the Developing Neocortex
Source: PLoS Biol. 2005 Jul 26;3(8):e272. doi: 10.1371/journal.pbio.0030272 (PMC1180514; doi:10.1371/journal.pbio.0030272)
Supplement: Supplementary file 1 [file pbio.0030272.sg002.pdf]

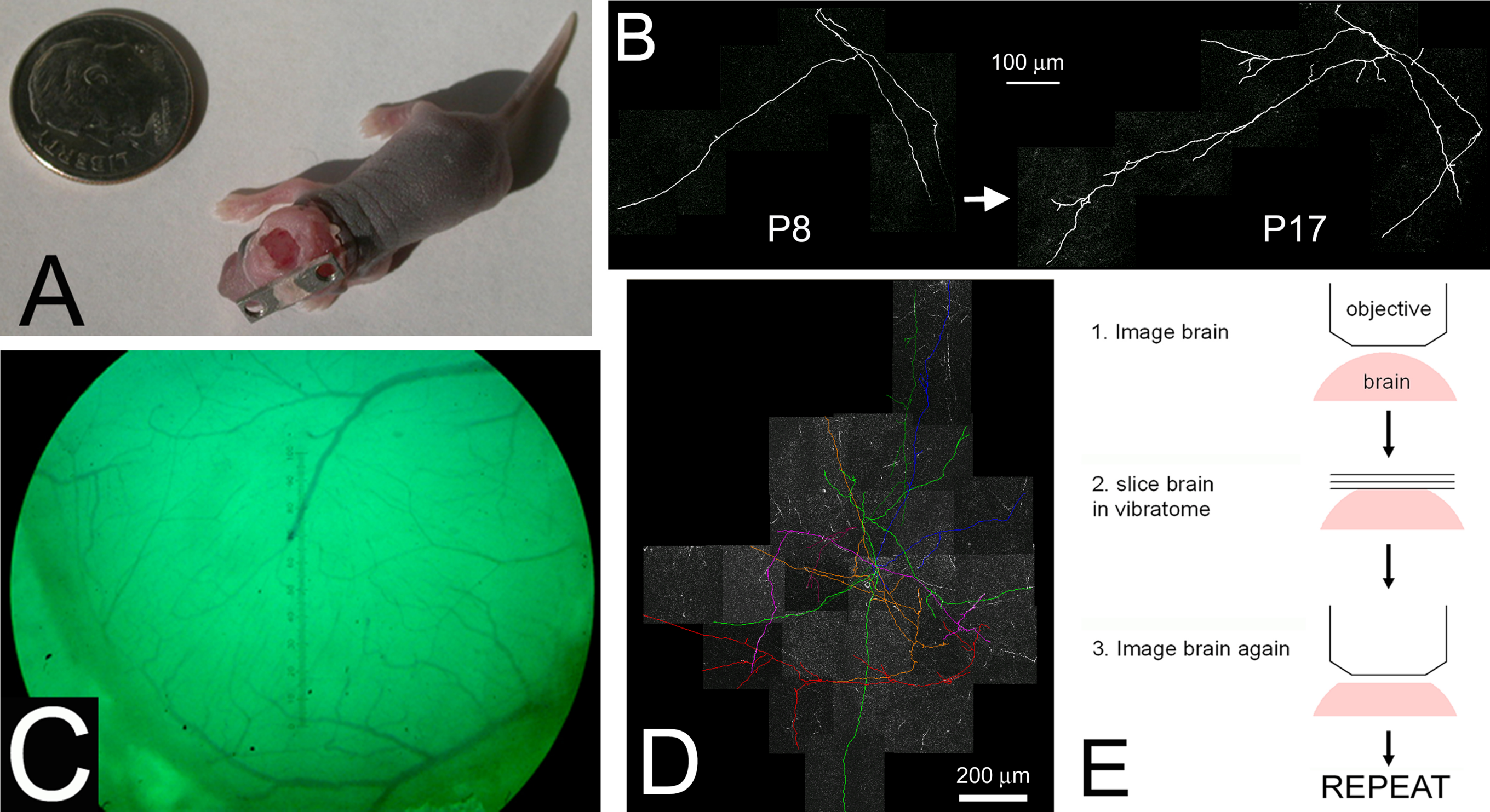
A
B
100 µm
P8
P17
C
D
200 µm
E
objective
1. Image brain
brain
2. slice brain
in vibratome
3. Image brain again
REPEAT
