# Supplementary material for: Diverse Modes of Axon Elaboration in the Developing Neocortex
Source: PLoS Biol. 2005 Jul 26;3(8):e272. doi: 10.1371/journal.pbio.0030272 (PMC1180514; doi:10.1371/journal.pbio.0030272)
Supplement: Supplementary file 2 [file pbio.0030272.sg003.pdf]

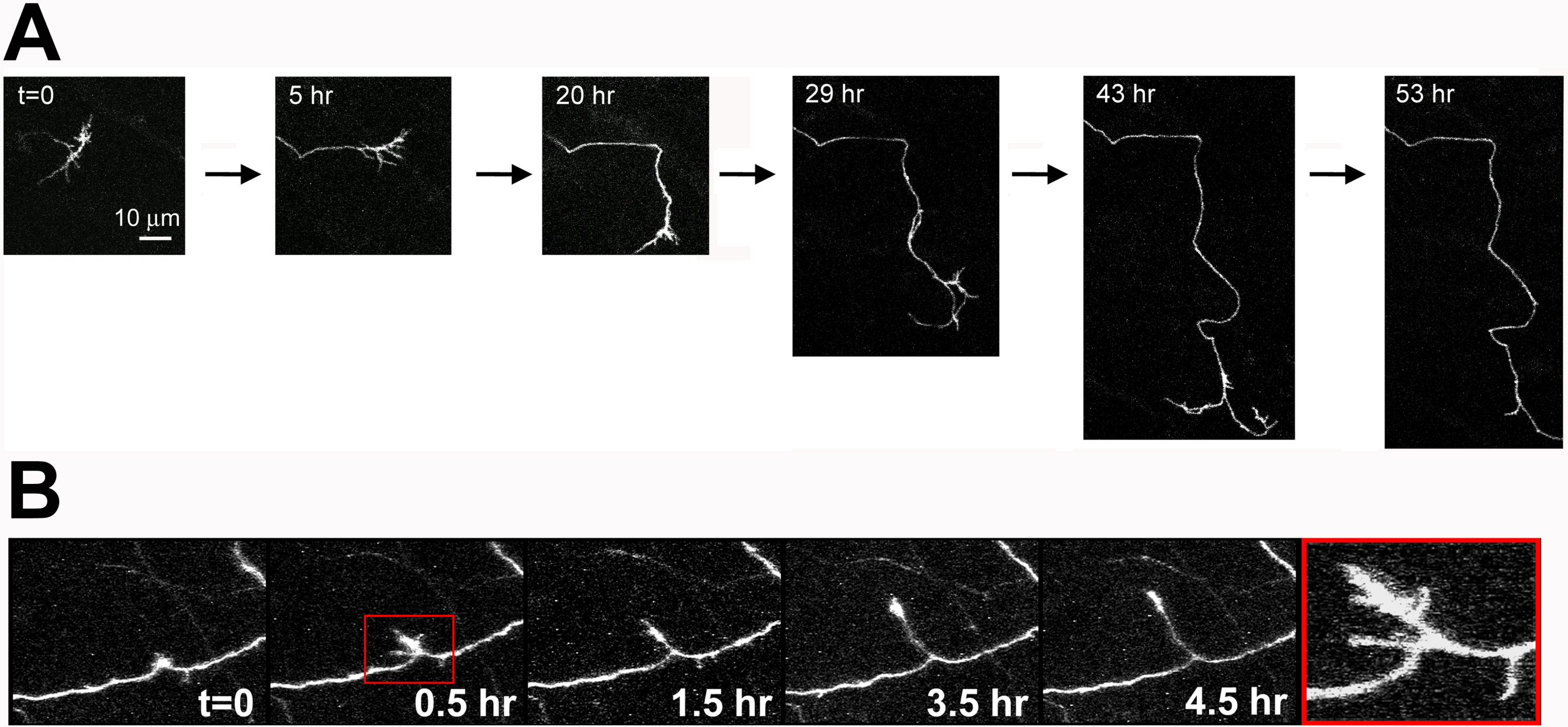
A
t=0
10 µm
5 hr
20 hr
29 hr
43 hr
53 hr
B
t=0
0.5 hr
1.5 hr
3.5 hr
4.5 hr
