# Supplementary material for: Diverse Modes of Axon Elaboration in the Developing Neocortex
Source: PLoS Biol. 2005 Jul 26;3(8):e272. doi: 10.1371/journal.pbio.0030272 (PMC1180514; doi:10.1371/journal.pbio.0030272)
Supplement: Supplementary file 3 [file pbio.0030272.sg004.pdf]

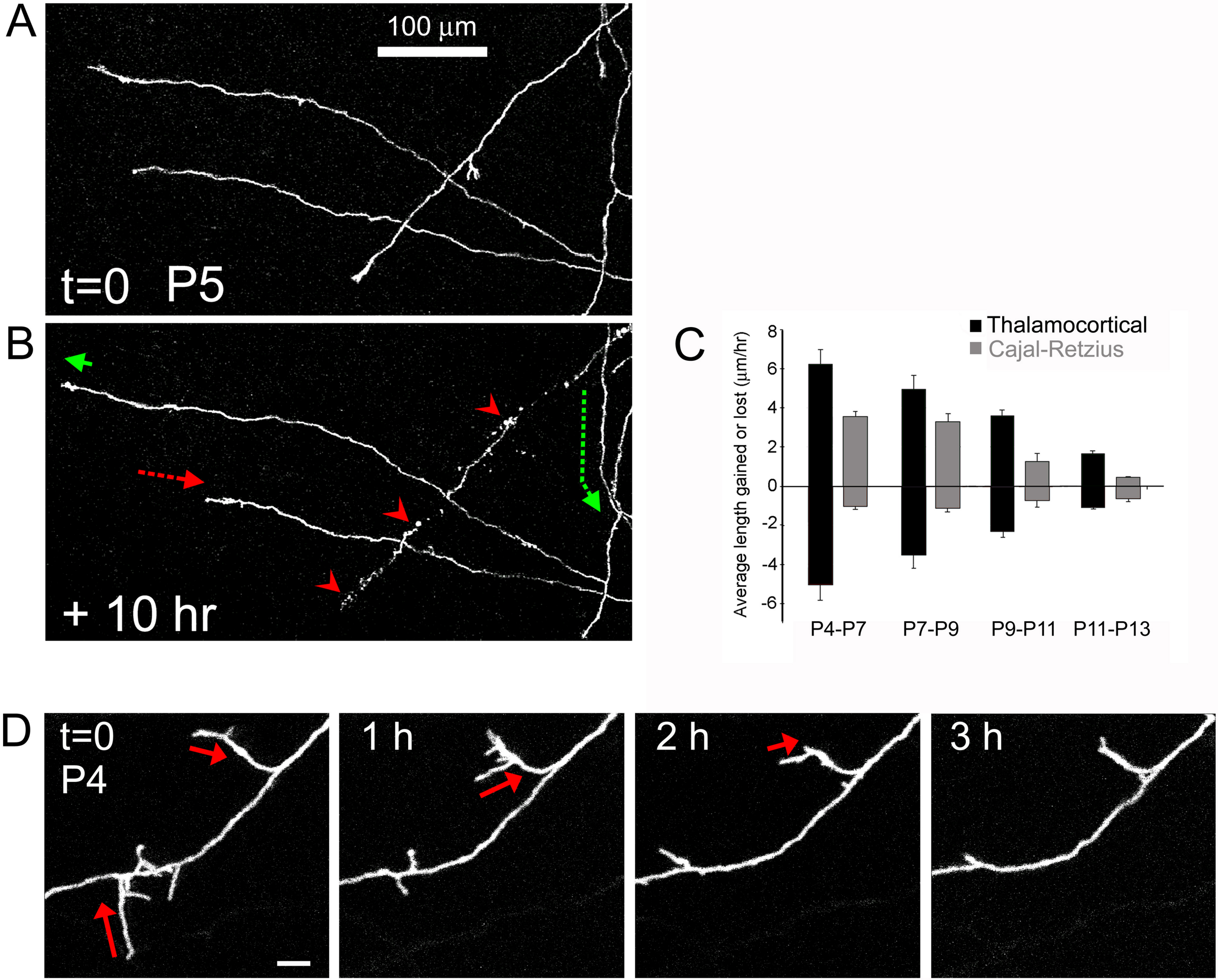

A
100 µm
t=0 P5
B
+ 10 hr
C
Thalamocortical
Cajal-Retzius
Average length gained or lost (µm/hr)
8
6
4
2
0
-2
-4
-6
P4-P7
P7-P9
P9-P11
P11-P13
D
t=0
P4
1 h
2 h
3 h
