# Supplementary material for: Diverse Modes of Axon Elaboration in the Developing Neocortex
Source: PLoS Biol. 2005 Jul 26;3(8):e272. doi: 10.1371/journal.pbio.0030272 (PMC1180514; doi:10.1371/journal.pbio.0030272)
Supplement: Supplementary file 4 [file pbio.0030272.sg005.pdf]

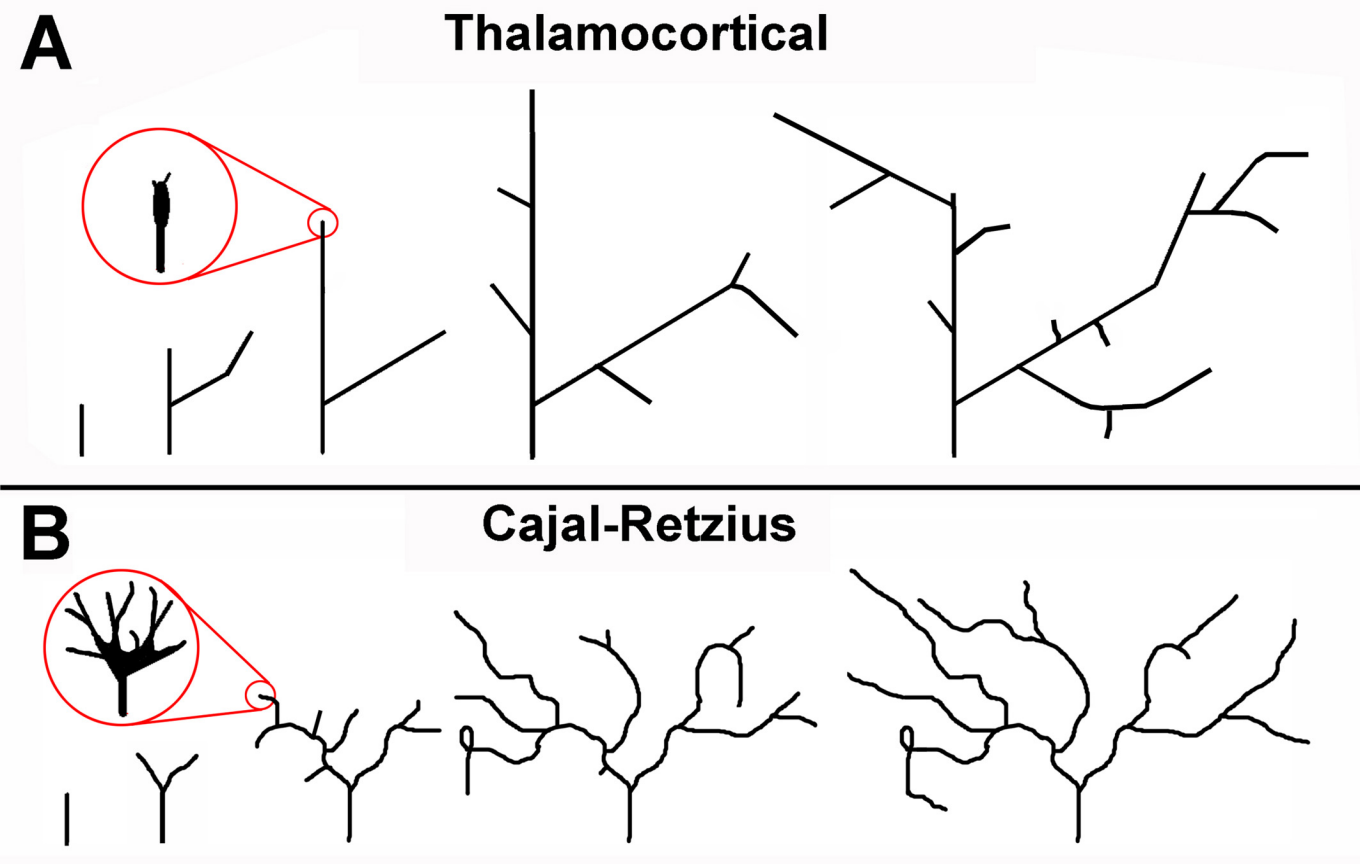
A
Thalamocortical
B
Cajal-Retzius
