# Supplementary material for: Diverse Modes of Axon Elaboration in the Developing Neocortex
Source: PLoS Biol. 2005 Jul 26;3(8):e272. doi: 10.1371/journal.pbio.0030272 (PMC1180514; doi:10.1371/journal.pbio.0030272)
Supplement: Supplementary file 5 [file pbio.0030272.sg006.pdf]

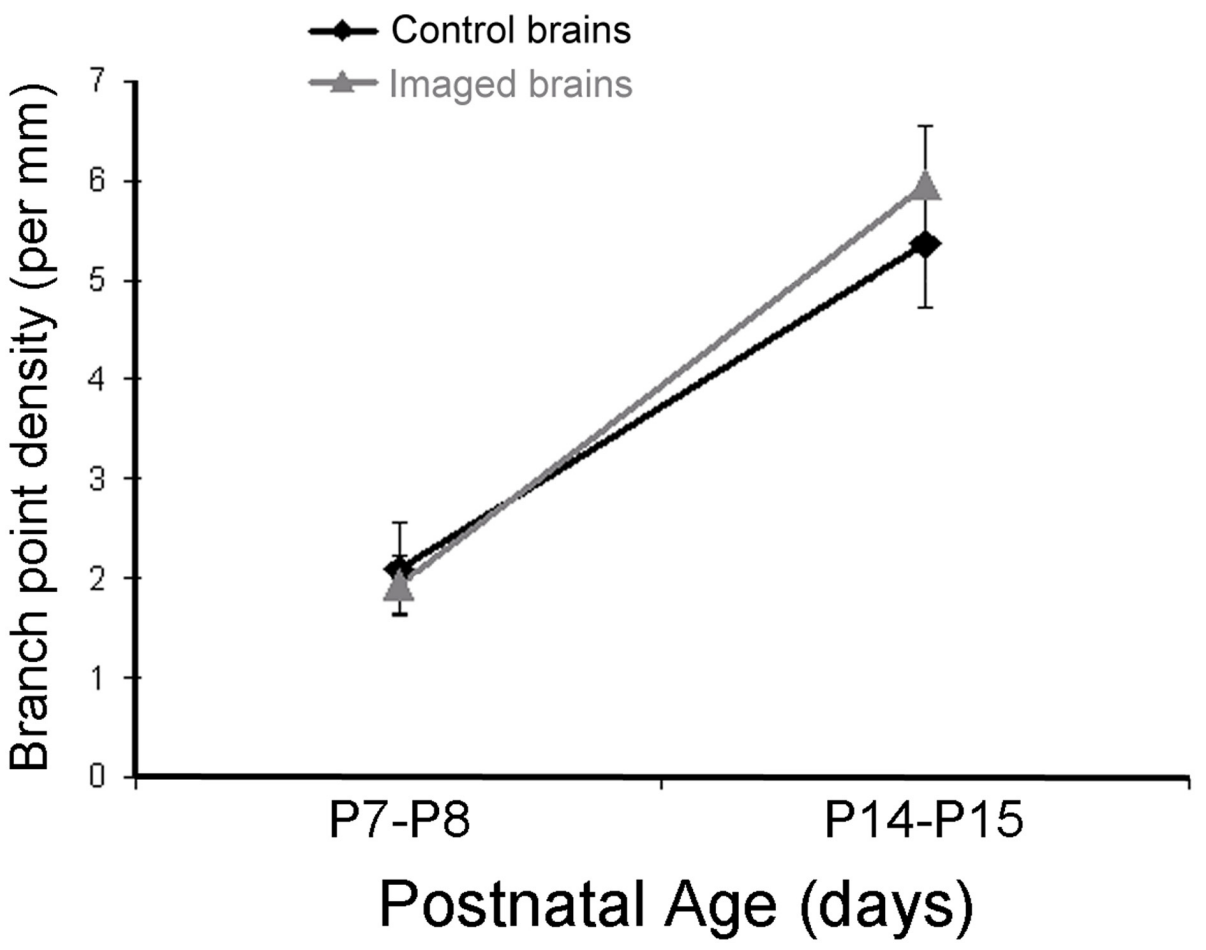

Control brains
Imaged brains
Branch point density (per mm)
0
1
2
3
4
5
6
7
P7-P8
P14-P15
Postnatal Age (days)
